# Engineering and Scaling the Spontaneous Magnetization Reversal of Faraday Induced Magnetic Relaxation in Nano-Sized Amorphous Ni Coated on Crystalline Au

**DOI:** 10.3390/ma9060426

**Published:** 2016-05-28

**Authors:** Wen-Hsien Li, Chi-Hung Lee, Chen-Chen Kuo

**Affiliations:** Department of Physics, National Central University, Jhongli 32001, Taiwan; k010211@yahoo.com.tw (C.-H.L.); s751734@hotmail.com (C.-C.K.)

**Keywords:** Au/Ni core/shell nanoparticle, amorphous Ni nanoparticle, Faraday induction, remanent magnetization, time relaxation

## Abstract

We report on the generation of large inverse remanent magnetizations in nano-sized core/shell structure of Au/Ni by turning off the applied magnetic field. The remanent magnetization is very sensitive to the field reduction rate as well as to the thermal and field processes before the switching off of the magnetic field. Spontaneous reversal in direction and increase in magnitude of the remanent magnetization in subsequent relaxations over time were found. All of the various types of temporal relaxation curves of the remanent magnetizations are successfully scaled by a stretched exponential decay profile, characterized by two pairs of relaxation times and dynamic exponents. The relaxation time is used to describe the reduction rate, while the dynamic exponent describes the dynamical slowing down of the relaxation through time evolution. The key to these effects is to have the induced eddy current running beneath the amorphous Ni shells through Faraday induction.

## 1. Introduction

Magnetic materials in the form of nano-sized particles or amorphous structures can generate many novel behaviors. In particular, memory and aging effects that reflect the characteristics of slow spin dynamics [1,2] have been observed [3,4,5,6,7,8] in magnetic nanoparticles (NPs). The memory effect in magnetic systems refers to the development of magnetization that is sensitive to changes in external condition, whereas the aging effect reflects the strong dependence of the relaxation of the magnetization on the waiting time before relaxation begins. It is understood that strong local spin correlation is the key in understanding these behaviors. An isolated single spin domain NP can be treated as a superspin [9,10], where the magnetic behavior is expressed by the particle magnetic moment of several hundreds or thousands of Bohr magnetons. The magnetic relaxation of the superspin will collectively freeze at low temperatures, with the freezing temperature strongly dependent on the particle or domain size and concentration [11,12,13,14]. An assembly of loosely packed magnetic NPs is currently described as a superparamagnetic system [15,16]. Turning on the interparticle interaction by closing up the interparticle separation drives the assembly to become a disordered system, with random anisotropy and completion of interparticle interaction. The broad distribution of the relaxation time and the random distribution of the particle position in the magnetic NP assembly can be a good source for the development of slow spin dynamics [17,18]. On the other hand, the magnetic material in the amorphous structure has an extremely low, if not zero, magnetocrystalline anisotropy energy, so that the directions of hard and easy magnetizations are largely relaxed. The low coercivity, low hysteresis loss, high permeability, and high electrical resistivity have made amorphous ferromagnet technologically valuable for applications as a soft magnetic material. Until recently, studies made on the dynamical behavior of amorphous magnetic NPs have still been very limited.

Remanent magnetization that lasts for a considerable period of time, obtained by relaxing the strength of the applied magnetic field H*_a_*, has been observed in many systems [19,20,21,22]. However, the remanent magnetization is weak, frequently amounting to only a very small percentage of the initial magnetization prior to the H*_a_* being switched off. Upon turning off the H*_a_*, the magnetization from the slow spin dynamics as well as from the Faraday induction will appear in the remanent magnetization. The dynamical behaviors of the two components can be very different, but the latter is frequently screened, as it is much smaller in amplitude and mixed in direction with the former. In a previous report [23], we have documented that huge inverse Faraday magnetizations can be induced in nano-sized amorphous Ni deposited on crystalline Au. However, there was no conclusive evidence found for the coexistence of magnetizations from slow spin dynamics and from inverse Faraday induction. In this study, we design and fabricate a larger core/shell structure, with 2.5 nm thick amorphous Ni on the shell and 5 nm crystalline Au in the core (marked Au/Ni), which allows a large enhancement of the magnetization in amplitude from Faraday induction, and separates it in direction from the residual magnetization of the slow spin dynamics. The eddy current was induced in the crystalline Au NP to run beneath the amorphous Ni, so that the induced Faraday magnetization points in the opposite direction to H*_a_*. The induced Faraday magnetization could be 2.5 times larger in amplitude than the residual magnetization of the slow spin dynamics. Relaxations of the two magnetic components can be described satisfactorily by the same scaling law, each having a characteristic temporal exponent and a characteristic time constant. Spontaneous magnetization reversals were revealed in the time relaxation of the remanent magnetization.

## 2. Results

### 2.1. Isothermal and Isofield Magnetizations

**Figure 1:**
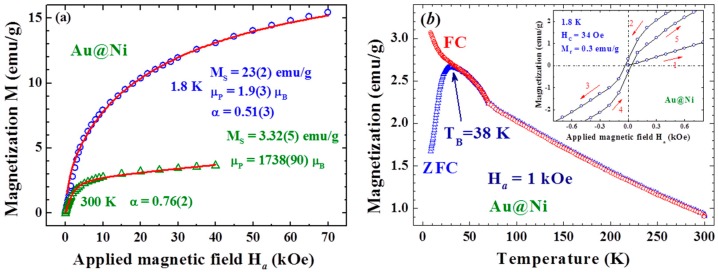
(**a**) isothermal magnetization curves M(H_a_) taken at two representative temperatures of 1.8 and 300 K, where the solid lines indicate the fits of the data to the reduced Lagnevin profile discussed in the text; (**b**) isofield magnetization curves M(T) measured in warming with H_a_ = 1 KOe, after FC (open circles) or ZFC (open triangles) from 300 to 2 K. The FC and ZFC curves depart from each other below 38 K, which marks the blocking temperature of the assembly. The inset shows the low field portion of the M(H_a_) loop taken at 1.8 K, revealing a coercivity of 34 Oe and a remanence of 0.3 emu/g.

The isothermal magnetization M(H_a_) curves can be satisfactorily described by the reduced Langevin profile, with a dynamic exponent α for the field parameter:

(1) $$M\left(H_{a}\right)={\text{ M}}_{S}\left(\cot x-\frac{1}{x}\right),\text{ x}\equiv \mu_{p}H_{a}^{\alpha }/k_{B}T$$

where M_S_ is the saturation magnetization, μ_p_ is the average particle moment, and k_B_ is the Boltzmann’s constant (Figure 1a). M_S_ reaches 23(2) and 3.32(5) emu/g at 1.8 and 300 K, respectively. Note that M_S_ of bulk Ni at 2 K is 58.6 emu/g. Critical exponents obtained at all temperatures studied depart greatly from one, resulting in α = 0.51(3) at 1.8 K and 0.76(2) at 300 K. It shows that the magnetic energy of the NP during measurement is smaller than the μ_p_H_a_ expected for systems with an instantaneous response to H_a_, but reveals behavior of a slow spin response as the wait time for each magnetization measurement is short. A larger value of α was obtained at a higher temperature, indicating that thermal agitation will result in faster magnetic responses. Magnetic hysteresis can be clearly seen in the M(H_a_) curves taken below 100 K, with a coercivity of H_C_ = 34 Oe and a low remanence of M_r_ = 0.3 emu/g at 1.8 K (inset to Figure 1b). The low M_S_ and M_r_ observed for the present assembly reflect the amorphous [24] and spin-glass [25,26,27] nature of the Ni in the Au/Ni NPs, whereas the extremely low H_C_ (34 Oe at 1.8 K) reflects the amorphous character of the Ni shell [24]. The temperature dependencies of the field-cooled (FC) and zero-field-cooled (ZFC) magnetizations taken at H_a_ = 1 kOe in warming depart from each other below 38 K, showing a blocking temperature of T_B_ = 38 K for the Au/Ni NPs at H_a_ = 1 kOe (Figure 1b).

### 2.2. Magnetic Relaxation

**Figure 2:**
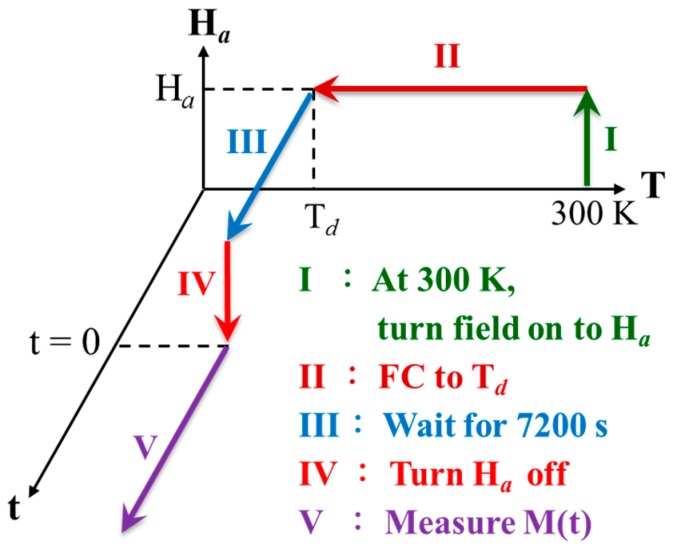
Schematics of the experimental processes in terms of temperature T, applied magnetic field H_a_, and time t in measuring the magnetic relaxation of the Faraday induced magnetization. The Roman letters and the arrows indicate the sequences of the processes.

**Figure 3:**
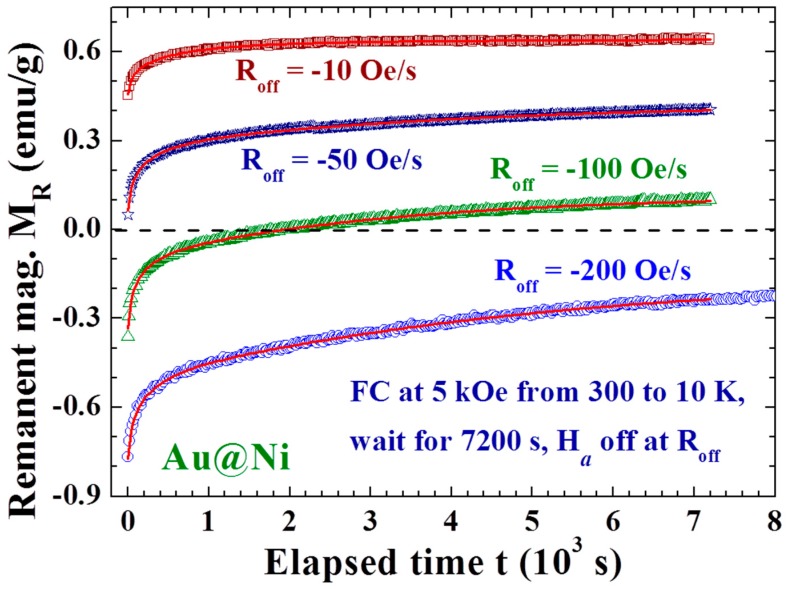
Time evolution of the remanent magnetization M_R_ recorded after field-cooling at H_a_ = 5 kOe from 300 to 10 K, followed by a 7200 s wait before turning the H_a_ off at rates of R_off_ = −200 Oe/s (open circles), −100 Oe/s (open triangles), −50 Oe/s (open stars), and −10 Oe/s (open squares). Spontaneous increases in the magnitude of M_R_ are seen in the R_0ff_ = −50 and −10 Oe/s curves. A spontaneous reversal in the direction of M_R_ appears in the R_off_ = −100 Oe/s curve.

The magnetic relaxation was measured after FC at a selected applied magnetic field H_a_ from 300 K to the designated temperature T_d_, followed by keeping the H_a_ on for a wait time t_w_ before switching it off at a selected field reduction rate R_off_ ≡ dH_a_/dt. The time of the first magnetization measurement performed right after H_a_ reaches zero is marked t = 0 for the study of temporal evolution of the remanent magnetization, as illustrated in Figure 2. It is interesting to see that the remanent magnetization M_R_ thus obtained at a low R_off_ (−10 and −50 Oe/s curves in Figure 3) evolves with time to larger values, rather than relaxing to smaller values. In addition, a higher R_off_ (−100 and −200 Oe/s curves in Figure 3) gives rise to a negative M_R_ at t = 0, marked M_R0_, which points in the opposite direction to H_a_ and relaxes to smaller values in magnitude. Clearly, Faraday induction generates an induced magnetization, which points in the opposite rather than in the same direction as H_a_ for the appearance of negative M_R0_. Remarkably, the M_R_ obtained with R_off_ = −100 Oe/s relaxes from pointing in the opposite direction to H_a_ at t = 0, crossing over at t ~ 1700 s to point in the same direction of H_a_ and continues to increase in magnitude. In addition, M_R_ relaxes to a sizable finite value, rather than to zero. This spontaneous reversal of the direction of the remanent magnetization together with the increase in magnitude after the reversal in the time evolution has not yet been reported.

**Figure 4:**
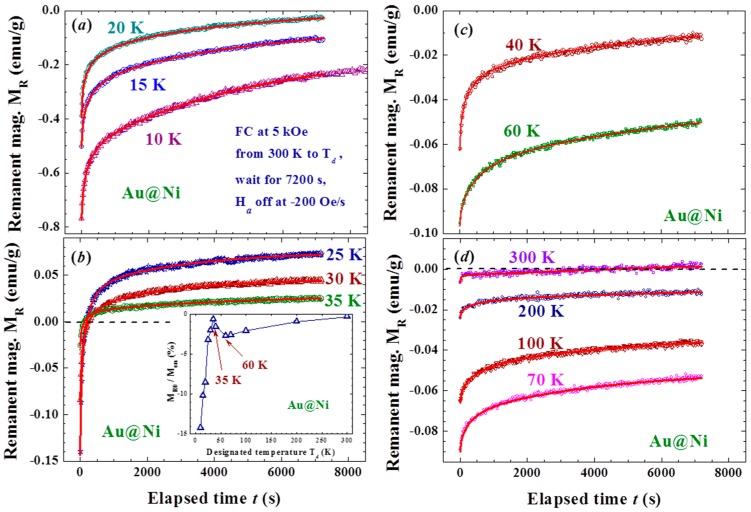
Time evolution of the remanent magnetization recorded after field-cooling at H_a_ = 5 kOe from 300 K to several representative temperatures covering 15 to 300 K, followed by 7200 s wait before turning the H_a_ off at a rate of −200 Oe/s (**a**–**d**). The solid lines indicate the fits of the data to the reduced exponential decay profile as discussed in the text. The inset to (**b**) shows the variations of the remanent magnetization obtained at t = 0, M_R0_, with temperature T_d_, revealing two turns at 35 and 60 K in the dependency of M_R0_ with T_d_. M_R0_ reaches ~15% of the initial magnetization M_on_ before H_a_ is switched off at 10 K.

**Figure 5:**
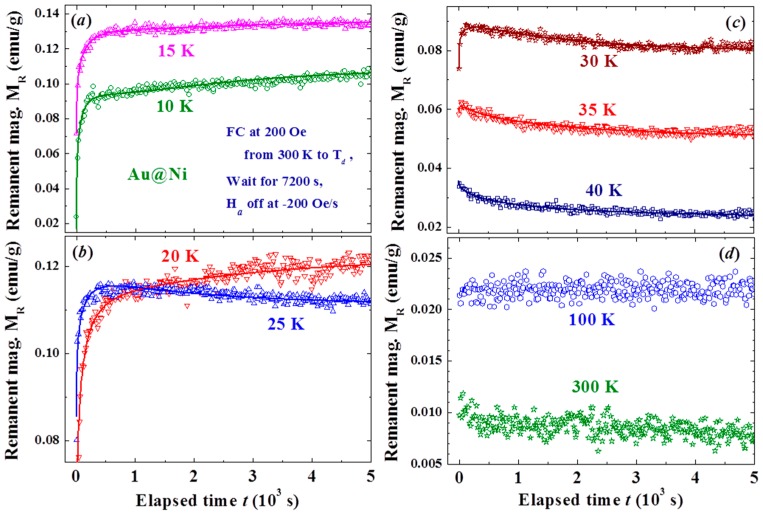
Time evolutions of the remanent magnetizations recorded after field-cooling at H_a_ = 200 Oe from 300 K to nine representative temperatures of (**a**) 10 and 15 K; (**b**) 20 and 25 K; (**c**) 30 to 40 K; and (**d**) 100 to 300 K, followed by a wait of 7200 s before turning the H_a_ off at a rate of −200 Oe/s. The solid lines indicate the fits of the data to the stretched exponential decay profile discussed in text.

**Figure 6:**
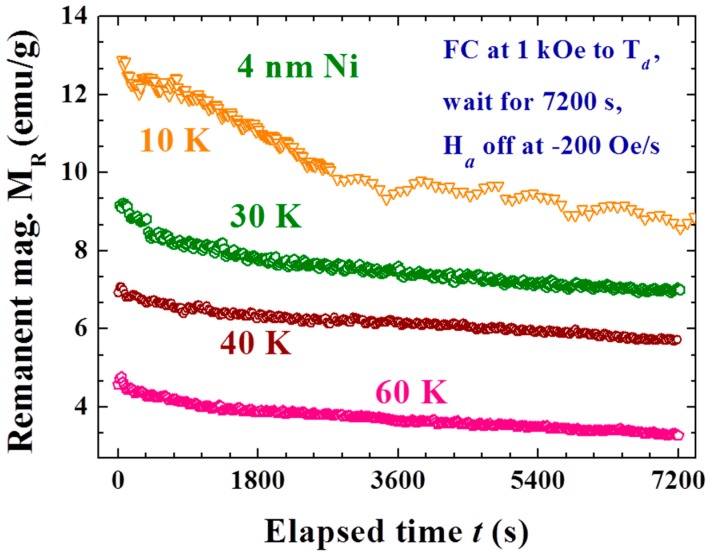
Time evolution of the remanent magnetization of the 4 nm Ni NP assembly recorded after field-cooling at H_a_ = 1 kOe from 300 K to various T_d_, followed by a wait for 7200 s before turning off the H_a_ at a rate of −200 Oe/s.

The effects are closely linked to T_d_, with a smaller M_R0_ obtained at a higher T_d_ (Figure 4a,b). Spontaneous magnetization reversals are revealed in the M_R_(t) curves generated when T_d_ lies between 25 and 35 K (Figure 4b). M_R0_ becomes larger in magnitude when generated at an even higher T_d_ of between 35 and 60 K (Figure 4c). A smaller M_R0_ is again generated when T_d_ becomes higher than 60 K (Figure 4d). The effect is greatly reduced but still clearly visible even with T_d_ = 300 K, where the magnetization reversal appears again at t ~ 4000 s. Two turns in the dependency of M_R0_ on T_d_ are revealed at 35 and 60 K (inset to Figure 4b). M_R0_ reaches ~15% of the initial magnetization M_on_ before H_a_ is switched off. Note that the magnetization at 5 kOe reaches only 22% of the saturation magnetization. Similar but weaker effects were also observed in the M_R_(t) curves generated with a weaker H_a_ of 1 kOe, even at 200 Oe. However, the M_R0_ generated with H_a_ = 200 Oe did not reach a negative value even with T_d_ = 10 K (Figure 5). It appears that the strength of the Faraday induction was strongly linked to M_on_ before the H_a_ was switched off. These interesting behaviors again reflect the sensitivity of the Faraday induction of the amorphous Ni in Au/Ni NPs to changes in the external disturbances, namely the appearance of the memory effect. The magnetic interaction between the spin domains within each individual NP must play a decisive role in the present observations. On the other hand, there was no inverse magnetization that appeared in the M_R_(t) curves generated using the same induction process on a 4 nm crystalline Ni NP assembly (Figure 6). It shows that the observed inverse Faraday magnetization is of material character in origin but is not linked to the small residual field that may appear on turning the H_a_ off.

## 3. Discussion

### 3.1. Scaling Parameters

The time behavior of the magnetic relaxation of M_R_ can be understood by assuming that there are two competing magnetic components that appear in the M_R_: one where the magnetization points in the same direction as H_a_, and the other where the magnetization points in a direction opposite to H_a_. The residual magnetization, marked M_r_, linked to the slow spin dynamics of the NPs will point in the same direction as H_a_, giving rise to a positive magnetization. The induced magnetization, marked M_i_, generated from the Faraday induction points in the opposite direction to H_a_, giving rise to a negative magnetization. Both M_r_ and M_i_ are relaxing in magnitude but have their own characteristic profiles and relaxation time. The increase or decrease of M_R_ over time evolution and the crossover from negative to positive M_R_ are the net results of completing M_r_(t) and M_i_(t) on temporal relaxation. In addition, M_R_ apparently relaxes to a finite value, marked M_0_, rather than to zero. The magnetic relaxation of the remanent magnetization after the applied magnetic field is switched off can then be expressed as M_R_(t) = M_0_ + M_r_(t) + M_i_(t).

**Figure 7:**
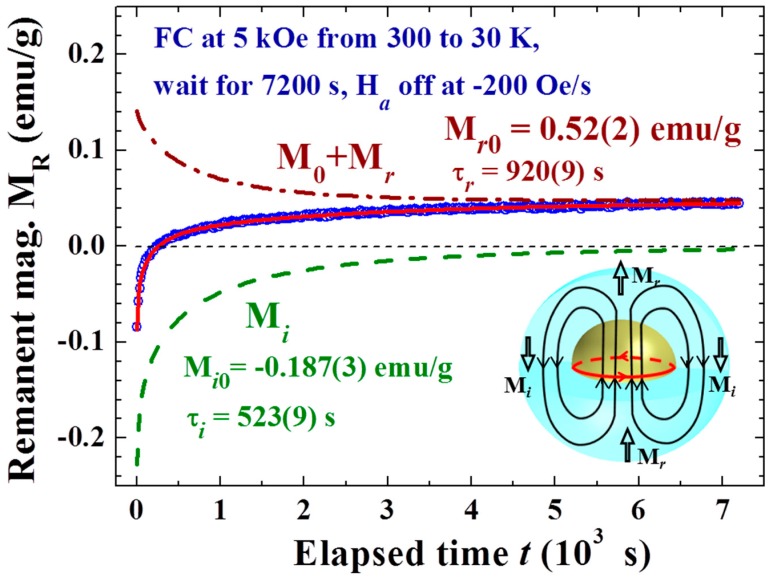
Time evolution of the remanent magnetization recorded after field-cooling at H_a_ = 5 kOe from 300 to 30 K, followed by a wait for 7200 s before turning off the H_a_ at a rate of −200 Oe/s. The solid lines indicate the fit of the data to the reduced exponential decay profile discussed in text. The dot-dashed curve in the upper panel indicates the final magnetization M_0_ plus the residual magnetization M_r_. The dashed curve in the lower panel indicates the induced magnetization M_i_. The inset shows a schematic representation of the Faraday induced current and magnetic field shortly after H_a_ reaches zero, where the eddy current in the Ni shell has dissipated, while that in the Au core (with a lower resistivity) is still circulating to produce an inverse magnetic field H_i_.

Upon turning the H_a_ off, the induced Faraday electric field will trigger eddy currents in the Ni shell as well as in the Au core. The flux changes in the amorphous Ni are considerably larger than the changes in the crystalline Au, so that the induced Faraday current is led to compensate for the flux changes in the Ni shell. Taking the H_a_ as pointing in the upward direction, the eddy currents will circulate counterclockwise on the horizontal plane. The eddy currents in the crystalline Au cores dominate over and last for a longer time than those in the amorphous Ni shells, since the resistivity of the amorphous Ni is significantly higher. The counterclockwise circulating eddy current in the Au core beneath the Ni generates an induced magnetic field that is opposite to the H_a_ on either side of the Au NPs, which aligns the nearby Ni superspins downward to produce an induced magnetization that points in a direction opposite to H_a_, whereas the induced magnetic field at the top and bottom of the Au NPs points upward, which acts to align the nearby Ni superspins to produce a magnetization that points in the same direction as H_a_ (inset to Figure 7). The latter component is mixed in direction and is inseparable from the residual magnetization from slow spin dynamics of the NPs.

The temporal profiles of the magnetic relaxation can be described (solid curves in Figure 3, Figure 4 and Figure 5) by assuming that M_r_(t) and M_i_(t) decay exponentially with time, but each has a characteristic dynamic exponent b together with a characteristic relaxation time τ:

(2) $$M_{r}{(t)=M}_{\text{r0}}\text{exp}\left\{-{\left(\frac{t}{\tau_{r}}\right)}^{b_{r}}\right\}{\text{ and M}}_{i}{(t)=M}_{\text{i0}}\text{exp}\left\{-{\left(\frac{t}{\tau_{i}}\right)}^{b_{i}}\right\}$$

where M_r0_, b_r_, and τ_r_ are the full magnitude, dynamic exponent, and relaxation time of the residual magnetization, respectively; and M_i0_, b_i_, and τ_i_ are those of the induced magnetization. The dynamic exponent b is used here to reflect the changes of relaxation rate with the evolution of time. A value for b of smaller than one indicates that the relaxation is slowing down, whereas a value for b of larger than one indicates the relaxation is speeding up. Figure 7 illustrates, as an example, the individual M_0_ + M_r_(t) and M_i_(t) components obtained from such a fit to the magnetic relaxation M_R_(t) observed in the process listed in the plot. A negative value for M_R0_ is obtained right after the induction operation, showing that the magnitude of M_i0_ is larger than that of M_r0._ It is the much faster decay rate of M_i0_ (characterized by τ_i_ = 523 s) than that of M_r0_ (characterized by τ_r_ = 920 s) that results in the magnitude of the negative component becoming smaller than that of the positive component at t = 250 s to reveal magnetization reversal and M_R_ continues to increase at later times. It is remarkable to see that all the various types of magnetic relaxation M_R_(t) curves observed in this study can be described by such a scaling law, where the scaling parameters reflect the internal magnetic characteristic of the NP assembly.

**Figure 8:**
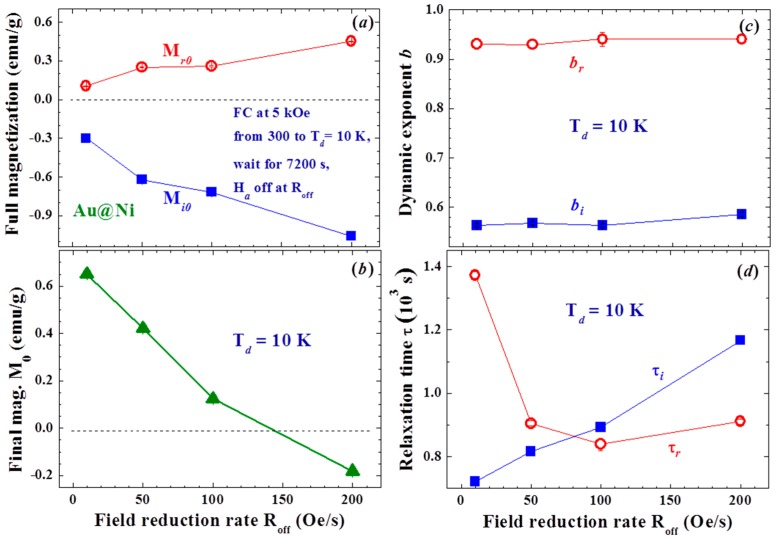
(**a**) variations of full residual magnetization M_r0_ and full induced magnetization M_i0_ with field reduction rate; (**b**) variations of final magnetization M_0_ after long relaxation with field reduction rate; (**c**) variations of dynamic exponent of residual magnetization b_r_ and induced magnetization b_i_ with field reduction rate; and (**d**) variations of relaxation time of residual magnetization τ_r_ and induced magnetization τ_i_ with field reduction rate.

Both M_r0_ and M_i0_ are strongly affected by the field reduction rate R_off_ (Figure 8a). M_r0_ is the expected compensation from Faraday induction plus the residual component from the slow spin responses that occur upon turning the H_a_ off, whereas M_i0_ is the additional component revealed in this study. Surprisingly, M_i0_ is significantly larger in magnitude than M_r0_, and the difference is enlarged at higher R_off_, so that a negative M_0_ is obtained at a high R_off_ (Figure 8b). It is revealed that larger portions of the Ni spin domains experience a Faraday magnetic field that points in a direction opposite rather than parallel to H_a_. The dynamic exponents b_r_ and b_i_ are essentially not affected by R_off_ (Figure 8c). The b_r_ obtained from the fits is nearly 1 (b_r_ ~ 0.94), showing that there is only a slight slowdown of the relaxation of M_r_ through the evolution of time at T_d_ = 10 K. On the other hand, b_i_ departs greatly from 1 (b_i_ ~ 0.56), showing that M_i_ relaxes at a significantly slower rate with the evolution of time. It is very unlikely that the slowing down of the magnetization relaxation is linked to the growth of the magnetic correlation length, as is frequently expected, but reflects the longer and longer time it can take for domain superspins to randomly flip. This can be understood if assuming that there are wide dimensions of ferromagnetic spin domains in the amorphous Ni shell of each NP. The mean time between two random flips of the domain superspin, known as the Neel relaxation time, is longer for a larger spin domain. The magnetic relaxation is dominated by larger spin domains at later times, giving rise to the slowing down of the relaxation. The relaxation times are on the order of 10^3^ s. A longer relaxation time τ_i_ for M_i_ is seen at higher R_off_ (filled squares in Figure 8d), reflecting that the M_i_ generated at a higher R_off_ relaxes at a slower rate. Interestingly, M_r_ displays the opposite trend as τ_r_ decreases with increasing R_off_ (open circles in Figure 8d). This is a direct result of a higher R_off_ that will generate more large spin domains for M_i_ that in turn limits the sizes of the spin domains of M_r_. The extremely long relaxation times of M_i_ and M_r_ reflect the existence of strong coupling among the magnetic domains in each individual NP. The NPs are only very loosely packed with a packing fraction of f = 10%, so that interparticle interaction is limited. Here, f indicates the ratio of the mass densities of the NP assembly to that of its bulk counterpart. The appearance of the spin-glass type memory effect in the Au/Ni NPs could be due to the spin frustration of amorphous Ni on the shell [27,28,29].

### 3.2. Thermal Effects

**Figure 9:**
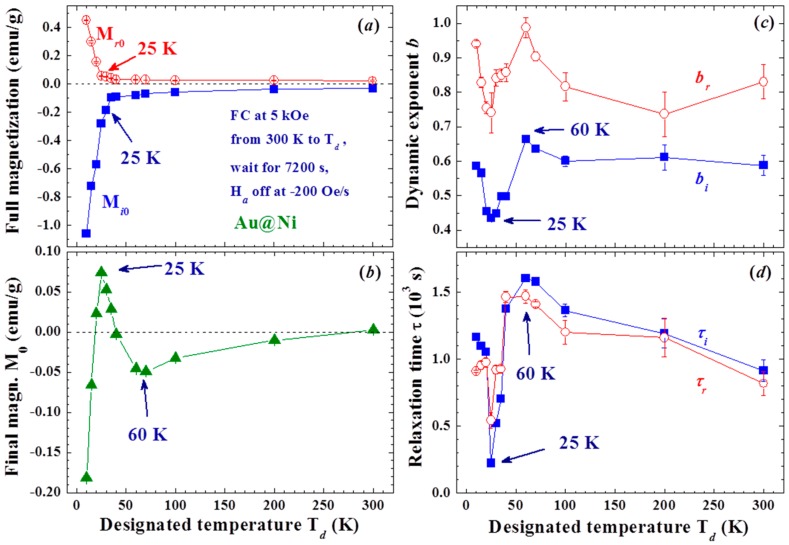
(**a**) variations of full residual magnetization M_r0_ and full induced magnetization M_i0_ with T_d_; (**b**) variations of final magnetization M_0_ after long relaxation with T_d_; (**c**) variations of dynamic exponents of residual magnetization b_r_ and induced magnetization b_i_ with T_d_; and (**d**) variations of relaxation time of residual magnetization τ_r_ and induced magnetization τ_i_ with T_d_.

**Figure 10:**
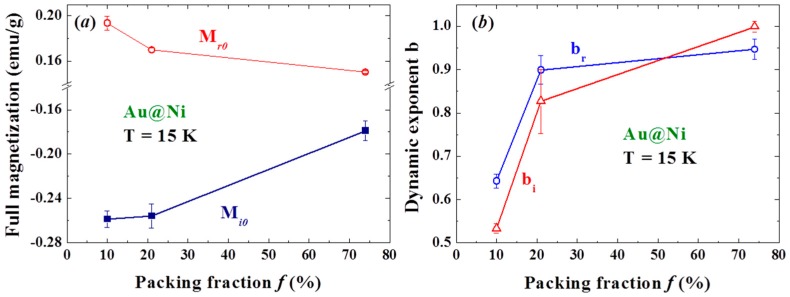
Variations of (**a**) full induced magnetization M_i0_ and full residual magnetization M_r0_; and (**b**) dynamic exponents b_i_ and b_r_ with the packing fraction of the NP assembly.

The scaling parameters of the magnetic relaxation were found to be sensitive to T_d_. Although the strengths of the induced as well as the residual magnetizations are largely reduced above the blocking temperature (T_B_ = 18 K at H_a_ = 5 kOe for the present measurements of measurement time ~1 s), they are still visible even at 300 K (Figure 9a). It is known that the superspins in NPs are essentially locked from random flips below T_B_. The significant reductions of M_i0_ and M_r0_ from the unlocking of the thermal flips of the domain superspins show that the observed spin-glass-like effect is linked to the correlations within and likely between the spin domains in the amorphous Ni shell of each Au/Ni NP. Surprisingly, turning on the interparticle interactions (by more closely packing the NPs) weakens, rather than strengthens, M_i0_ and M_r0_ (Figure 10a), revealing that the effects are driven mainly by the interactions between the spin domains within each NP. This can be understood as a loss of the amorphous nature of the Ni shell from interparticle interaction that will weaken the induction from turning H_a_ off. Note that the coercivity and remanence of the f = 74% assembly increases to H_C_ = 258 Oe and M_r_ = 1.7 emu/g at 15 K.

Two distinct changes at 25 and 60 K are revealed in the T_d_-dependencies of the scaling parameters M_0_ (Figure 9b), b_i_ and b_r_ (Figure 9c), τ_i_ and τ_r_ (Figure 9d). M_0_ is the remanent magnetization after a long relaxation of M_i_ and M_r_. It contains two components, one from M_i_ and the other from M_r_, which are unfortunately inseparable in the analysis. The appearance of M_0_ shows that large spin-domains exist, where a higher thermal energy and/or much longer relaxation time are needed for randomly flips of the domain superspins. Negative values for M_0_ are obtained, except for a crossover to positive values between 20 and 40 K (Figure 9b). Apparently, there are more large spin domains in the M_i_ component than in the M_r_ component, but the situation is reversed in the narrow temperature window between T_B_ and 20 K above. This is totally unexpected, since the NPs experience an H_a_ of 5 kOe for 7200 s before experiencing a reducing H_a_ at −200 Oe/s for only 25 s. The changes in the rate of the slowdown of the relaxation on temporal evolution with T_d_ (Figure 9c) are closely connected with that of M_0_ (Figure 9b) and τ (Figure 9d). Interestingly, the T_d_-dependencies of the dynamic exponents and relaxation times display opposite trends below and above 25 K. It is difficult to attribute the reversals of the T_d_-dependencies of b and τ at 25 K to the changes of sign of the governing sources, since there is no transition that can be linked to this temperature regime. The differences can, nevertheless, be ascribed to the differences in the thermal characteristics of M_i_ and M_r_. Clearly, M_i_ dominates at low temperatures, with a higher degree of relaxation slowdown and a shorter relaxation time at a higher T_d_. On the other hand, M_r_ becomes revealed over M_i_ above 25 K, with a lower degree of relaxation slowing down and a longer relaxation time at a higher T_d_. The distinct differences between the thermal characters of M_i_ and M_r_ are obviously originated from the differences in the processes that create them, namely the appearance of aging effect. M_r_ is mainly created by the H_a_ with a wait time of 7200 s, more stable spin domains of less degree of spin fluctuations for M_r_ can be expected to reveal a dynamic exponent that is closer to 1. On the other hand, M_i_ is created by switching the H_a_ off over 25 s, the spin fluctuations of the domains in M_i_ can give rise to a higher degree of relaxation slowing down in time evolution. Note that the dynamic exponents increase to nearly 1 for b_i_ and b_r_ as f is increased to 74% (Figure 10b). Apparently, interparticle interaction weakens the observed memory and aging effects.

## 4. Materials and Methods

The X-ray diffraction measurements for structural investigation were performed on a Bruker D8 ADVANCE diffractometer (Billerica, MA, USA), employing the standard reflection geometry. Chemical analysis by means of energy dispersive X-ray spectroscopy (EDXS) was also performed to characterize the elemental composition of the sample. The EDXS spectra were taken with a HORIBA EX-220 detector (Kyoto, Japan) attached on a HITACHI S-4200 scanning electron microscope (SEM) (Kyoto, Japan), employing a standard setup to analyze 12 portions of the sample. The high-resolution transmission electron microscope (HRTEM) images were taken using a JEOL JEM-2100 (Tokyo, Japan), employing an acceleration voltage of 200 kV for a magnification of 3 × 10^5^ times. The atomic force microscope (AFM) images of the NPs were taken using a Nanoscope-III (Veeco Instruments Inc., Plainview, NY, USA) operated in tapping mode, where a noncontact technique, with the cantilever tip vibrating at a large amplitude to avoid trapping, was used to profile the sample surface for size analysis. To reduce aggregation of the NPs, the powder was shaken at 30 Hz for 5 min using a Vortex-Genie Mixer (Scientific Instruments Inc., Ringoes, NJ, USA) before it was packed into a thin nonmagnetic cylindrical holder for magnetization measurement. The holder, manufactured by Quantum Design (San Diego, CA, USA), produced a smooth temperature curve and a background signal that was less than 1% of the signal from the sample. The initial packing fraction f of the NP assembly was ~10%, which indicates the ratio of the mass densities of the assembly to that of its bulk counterpart. Magnetization measurements were performed on a Physical Property Measurement System (San Diego, CA, USA), employing the standard setups and the linear mode for magnetic field charging and discharging at a selective rate. Low field charging/discharging rates are used to minimize the possible effects from overshooting of the magnetic field. The magnetization was measured by detecting the change in the magnetic flux as the sample was removed from the sensing core.

The Ni/Au NPs were fabricated employing the gas-condensation method, using a chamber equipped with two decoupled evaporation sources for separate evaporation of Ni or Au. High-purity Au/Ni spheres (~0.3 g each, 99.99% pure and ~2 mm in diameter) were heated separately using a current source of 77/95 A, and were evaporated at a rate of 0.05 Å/s in an Ar atmosphere using a pressure of 3.2 torr. Note that at a low evaporation rate, the key to control the mean particle diameter is the Ar pressure in the chamber during evaporation, which controls the times of collisions experienced by the evaporated atoms before they reach the collector. Note that the evaporated atoms will not have enough kinetic energy to reach the collector when a high chamber pressure is employed. The evaporated particles were collected on a non-magnetic SS316 stainless steel plate placed 20 cm above the evaporation source and maintained at 77 K. After restoration to room temperature, the NPs, which were only loosely attached to the collector, were stripped off. The samples thus obtained were in powdered form and consisted of a macroscopic amount of individual NPs. There was no substrate or capping molecules on the NPs. The resultant powder was no longer gold yellow but dark black, indicating that the absorption bands of the powders have blue shifted to the invisible region, as is the case for most metallic NPs. The NPs were kept in an Ar atmosphere at all times during evaporation, sample collection, and encapsulation into holder.

**Figure 11:**
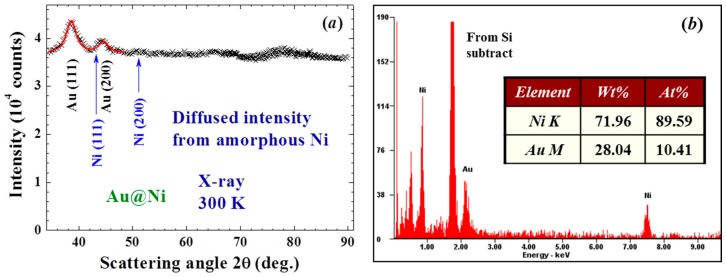
(**a**) X-ray diffraction pattern of the Au/Ni nanoparticles taken at room temperature, revealing a series of diffraction peaks associated with face-centered cubic Au. The solid curves indicate the calculated profiles for 5 nm Au. The arrows indicate the expected positions for face-centered cubic Ni; (**b**) a representative EDXS spectrum, where signals from Ni are dominating to reveal an atomic ratio of Ni:Au = 89.6:10.4 for this spatial region of the assembly.

**Figure 12:**
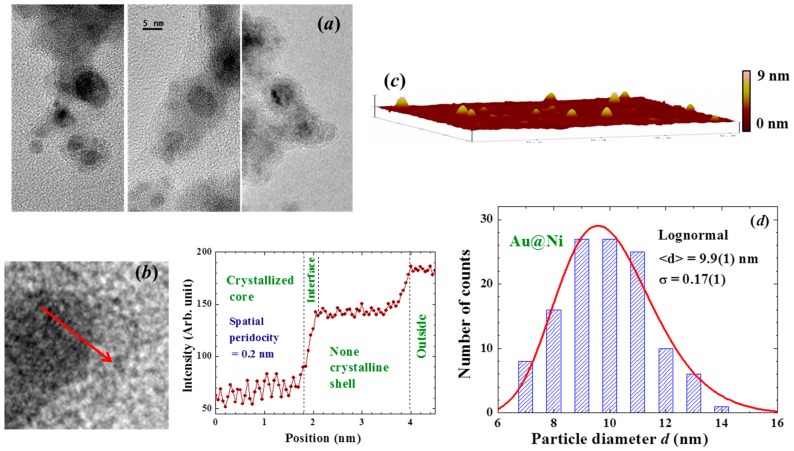
(**a**) representative HRTEM images of the Au/Ni NP, revealing the spherical core/shell structure of the NP; (**b**) enlarged HRTEM images focused in the interface regime, revealing a lattice periodicity fringe for the atoms in the core, but no spatial periodicity may be identified for the atoms on the shell; mean TEM intensity of the Au/Ni NP in the interface regime; (**c**) representative AFM images of the Au/Ni NP; and (**d**) size distribution obtained from the AFM and HRTEM images. The solid line indicates the results of the fit to a lognormal function, giving a mean particle diameter of 9.9 nm.

**Figure 13:**
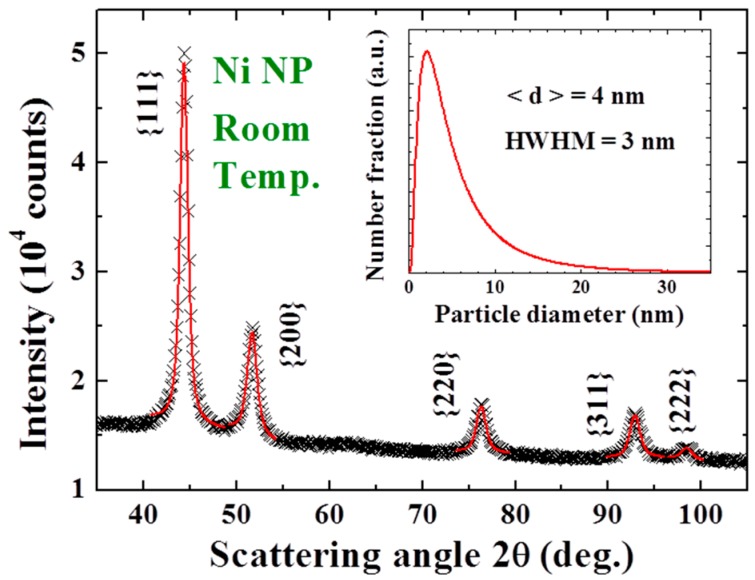
X-ray diffraction pattern of the Ni nanoparticle assembly taken at room temperature, revealing a series of diffraction peaks associated with face-centered cubic Ni. The solid curves indicate the calculated profiles for 4 nm Ni with the size distribution shown in the inset.

X-ray diffraction patterns, AFM and HRTEM images, and EDXS spectra were all taken to characterize the samples. No obvious differences were found in the X-ray diffraction patterns taken from different portions of the sample. The X-ray diffraction pattern of each portion of the sample reveals a series of broad but well-defined diffraction peaks from crystallized face-centered cubic (fcc) Au together with strong incoherent diffused intensity distributed over the entire pattern, and noticeable but very weak diffraction peaks associated with crystallized fcc Ni (Figure 11a). The expected positions for the Ni(111) and for the Au(200) reflections separate by 0.8 degrees in scattering angle. The widths of the peak at 38.6 and 44.6 degrees are nearly the same together with that the Ni(200) reflection is barely revealed (Figure 11a), indicating that the peak at 44.6 degree is contributed mainly from the Au(200) reflection. Elemental analysis using EDXS spectra taken from 12 different portions of the assembly gives an atomic ratio of Ni:Au = 90(2):10(1). A representative EDXS spectrum is shown in Figure 11b. It appears that the strong diffused intensity in the diffraction pattern is mainly from the amorphous Ni, and the main component of the NPs is indeed the noncrystalline Ni atoms. The HRTEM images (Figure 12a) display a spherical core/shell structure for the NPs, where the images of the cores are considerably darker than those of the shells. Lattice-fringe with a spatial periodicity of 0.2 nm, which marked the Au (200) lattice planes, is revealed in the core of the NPs, but no spatial periodicity can be identified on the shell (Figure 12b). It appears that crystallinity of the Ni atoms on the shell is limited if not nothing at all. The NPs thus have a core/shell structure with crystalline Au in the core covered with an amorphous Ni shell. Size analysis based on the AFM (Figure 12c) and HRTEM images reveals that particle sizes of the NP assemblies can be described using a lognormal distribution, with a mean particle diameter of 9.9(1) nm and a standard deviation of 0.17(1) (Figure 12d). The mean particle diameter of the crystalline Au was determined by fitting the diffraction peaks to the diffraction profiles of finite sized particles, assuming a lognormal size distribution for the NP assembly. The mean particle diameter thus determined for the Au cores is 5 nm. Knowing the size of the Au core and the atomic ratio between Ni and Au, we estimate that the Au/Ni NP consists of 5 nm fcc Au at the core covered by a 2.5 nm thick amorphous Ni shell, resulting in a mean particle diameter of 10 nm for the Au/Ni, which agrees well with what was obtained from the AFM and HRTEM images. The X-ray diffraction pattern of the Ni NP assembly reveals a series of broad but well-defined diffraction peaks from crystallized face-centered cubic Ni (Figure 13). No traces of oxidation phases or elements other than Ni may be identified from the diffraction patterns. The mean particle diameters are determined by fitting the diffraction peaks to the diffraction profiles of finite sized particles [30]. The solid curves in Figure 13 indicate the calculated pattern assuming a log-normal size distribution with a mean particle diameter of 4 nm and a half-width-at-half-maximum of 3 nm, as shown in the inset to Figure 13.

## 5. Conclusions

The key components for inducing large inverse magnetization include the use of: (1) a magnetic material in its amorphous form to relax the hard magnetic axis for easy moment alignment, less magnetic anisotropic loss, and formation of ferromagnetic spin domains; (2) a conducting material with low resistivity that will support an induction current flowing beneath the magnetic material to generate a Faraday magnetic field that points in the opposite direction to the applied magnetic field; and (3) a nano-sized structure to provide more individual induction currents for effect enhancement. Large remanent magnetization that points in the opposite direction to the applied magnetic field can thus be generated in a nano-sized core/shell structure of Au/Ni by turning off the applied magnetic field. A spontaneous reversal in direction and increase in the magnitude of the remanent magnetization during the subsequent relaxation over time were found. Two magnetic components that point in the opposite directions were realized in the remanent magnetization. Their relaxations through time are successfully scaled by a relaxation time to describe the reduction rate together with a dynamic exponent to describe the dynamical slowing down of the relaxation with the evolution of time. Higher induction can be anticipated when an even higher field reduction rate is used. It would be interesting to see the effects of using a superconducting core to generate the inverse magnetization.

Finally, we remark that oxidation of nano-scaled Ni into NiO upon exposure to the air has been reported, where the spins in the NiO shell freeze into a disordered spin glass-like state below 40 K [31]. A logarithmic decay of magnetization with time [32], a power law dependence of the spin relaxation through time [32], and critical slowing down of the magnetic dynamic behavior [25] have all been observed in nano-sized spin NiO. It is possible that a thin layer of NiO exists on the surface of the present Au/Ni NP, even the NPs were kept in an Ar atmosphere at all times during evaporation, sample collection and encapsulation into holder. Crystallization of the atoms in the core is clearly revealed, but no sign of crystallization of the atoms on the shell may be found. The 2.5 nm shell in the Au/Ni NP can accommodate seven crystallized Ni unit cells without accounting for lattice mismatch at the Ni-Au interface (a = 0.352 nm for Ni and 0.401 nm for Au). It is unlikely that a well-crystallized lattice structure of NiO-Ni-Au will develop within a 2.5 nm length, if oxidation does occur. It is the surrounding magnetic clusters of Ni and/or NiO around the crystallized metal Au core that sense the Faraday induction at a high sensitivity and give rise to a large inverse Faraday response. It is the slow spin relaxation of the Ni and/or NiO clusters that give rise to a measureable magnetic relaxation for the current study.
